# What is the societal economic cost of poor oral health among older adults in the United States? A scoping review

**DOI:** 10.1111/ger.12548

**Published:** 2021-03-14

**Authors:** Shulamite S. Huang, Analia Veitz‐Keenan, Richard McGowan, Richard Niederman

**Affiliations:** ^1^ Department of Epidemiology and Health Promotion College of Dentistry New York University New York NY USA; ^2^ Department of Oral and Maxillofacial Pathology, Radiology and Medicine New York University New York NY USA

**Keywords:** economics, older adults, oral health, review

## Abstract

**Objective:**

To assess the state of the literature in the United States quantifying the societal economic cost of poor oral health among older adults.

**Background:**

Proponents of a Medicare dental benefit have argued that addressing the growing need for dental care among the US older adult population will decrease costs from systemic disease and other economic costs due to oral disease. However, it is unclear what the current economic burden of poor oral health among older adults is in the United States.

**Methods:**

We conducted a scoping review examining the cost of poor oral health among older adults and identified cost components that were included in relevant studies.

**Results:**

Other than oral cancer, no studies were found examining the economic costs of poor oral health among older adults (untreated tooth decay, gum disease, tooth loss and chronic disease/s). Only two studies examining the costs of oral cancer were found, but these studies did not assess the full economic cost of oral cancer from patient, insurer and societal perspectives.

**Conclusions:**

Future work is needed to assess the full economic burden of poor oral health among older adults in the United States, and should leverage novel linkages between medical claims data, dental claims data and oral health outcomes data.

## 1 INTRODUCTION

In 2030, almost 20% of US adults (80 million people) will be 65 years or older,^1^ will retain more teeth and have increasing dental needs^2^, ^3^ while facing fragmented access to dental care benefits.^4^, ^5^ Despite this, policymakers have not yet addressed whether the growing need for dental care necessitates improvements in dental care coverage and access among older adults. Proponents of rectifying the gaps in access to dental care coverage among older adults argue that increased costs to Medicare/Medicaid from providing dental care coverage would be offset by decreased costs from systemic disease. However, it is unclear what the current economic burden on individuals, insurers, and Medicare/Medicaid is due to poor oral health conditions among older adults. Whether researchers have quantified the current economic burden from poor oral health among older Americans is key in highlighting to policymakers whether efforts to decrease costs from poor oral health among older adults and to increase dental care coverage would be worthwhile.

Older Americans currently access dental care benefits in a piecemeal fashion. Dental care benefits are available to older Americans indirectly through employment benefits, post‐retirement dental benefits, spousal coverage or specific Medicare Advantage plans.^4^ Traditional Medicare (Parts A and B) does not include dental care benefits (CMS) outside of those that are important parts of covered medical procedures and hospital care resulting from complications of a dental procedure.^5^ Older Americans enrolled in Medicare Advantage plans (also known as Medicare Part C) may have access to dental benefits, but only 18% of Medicare beneficiaries in 2016 had access. Some older adults may access state Medicaid dental benefits if they are dually eligible for both Medicare and Medicaid, but Medicaid dental benefits for adults frequently fluctuate depending on the availability of state funds.^6^ As a result of the piecemeal dental care coverage across Medicare and Medicaid for older adults, nearly two‐thirds of all people on Medicare have no dental coverage.^5^

Limited coverage for dental care may be a major barrier to dental care among older adults and a reason for the high prevalence of untreated oral diseases (ie tooth loss, untreated caries and periodontal disease) among older adults.^7^ Due to limited insurance coverage for dental care at older ages, the share of dental expenditures paid for out‐of‐pocket increases with age.^7^ Though the proportion of older adults with expenses for dental visits has been increasing over time, indicating growing dental utilisation, the average expenses for a dental visit has also grown with time,^8^ while prevalence of edentulism has remained high (15% of those aged 65 to 74 years and 21.9% of those aged 75 years or older in 2009‐2010 were edentulous), indicating a failure of the dental care system.^7^ Untreated oral diseases can significantly impact the quality of life by restricting normal activities, disturbing sleep and causing pain.^7^, ^9^, ^10^, ^11^, ^12^, ^13^, ^14^, ^15^ Without access to care to resolve oral diseases, untreated oral disease will can lead to unplanned tooth loss or edentulism, which then relates to mastication and nutrition problems.^7^, ^9^, ^10^, ^11^, ^12^, ^13^, ^16^

However, some proponents for including a Medicare dental benefit go further, arguing that poor oral health increases the cost of managing systemic disease ^17^ and that increasing use of dental treatments can offset some health care costs. Multiple studies to date have found a strong association between poor general health and poor oral health,^7^, ^9^, ^14^, ^15^ while other studies have examined the impact of dental interventions on healthcare costs for systemic conditions.^18^, ^19^, ^20^, ^21^ However, a recent systematic review found that there is limited evidence for the impact of dental interventions on healthcare costs for chronic conditions,^19^ and the relationship between general health and oral health is likely bidirectional instead of causal.^7^

Poor oral health may impose additional economic costs beyond those directly related to disease. For instance, poor oral health may lead to productivity losses not only among an older individual with poor oral health, but also among that individual's caregivers because of increased difficulty chewing and receiving proper nutrition.^11^ It is currently unclear how much information exists to quantify the extent to which poor oral health among older adults increases not only the medical costs from treating systemic disease to the government, individuals, and the health system, but also travel and productivity costs for individuals with oral disease and those providing informal care.

The lack of information regarding the full economic burden of poor oral health among older adults impedes efforts to improve the oral health of ageing Americans. Due to constrained societal resources, not every health intervention that improves health can be implemented because of rising costs. Instead, policymakers and the popular press often use the economic costs of a variety of illnesses with epidemiological information to justify implementing intervention programmes aimed at reducing costs, improving health or both.^22^, ^23^ Medical researchers have increasingly used cost‐of‐illness studies to highlight the relative importance of disease in public policy discussions ^22^ and to assess the economic burden of illnesses to society. Cost‐of‐illness (COI) studies are separate from studies examining overall dental care expenditures among older adults, which may include expenditure on dental treatments that are not targeted at specific oral health conditions and do not include other cost components that may be relevant (ie costs from taking time off work, or productivity costs among families and friends). COI studies also should be distinguished from cost‐effectiveness and cost‐benefit analyses (CEA/CBA), which are used to understand which programmes or treatment should be implemented to optimise resource allocation. However, COI studies provide critical information for the costing component of CEAs and CBAs,^24^

Though studies of the economic burden of poor oral health among older adults exist in other countries, there is no single country that is easily generalisable to the United States. Prior literature has highlighted that not only are the prices and quantities used in the United States higher than in other countries, but other factors like quality and intensity likely vary between the United States and other countries. Additionally, the high prevalence of employer‐sponsored insurance in the United States compared to other countries leads to distortions in the price of insurance, the price of care, quantities demanded, services/treatments that are approved by the Federal Drug Administration and available for patients, and the value of care delivered that are not easily quantified. All these factors limit extrapolation from other countries’ analyses of economic and healthcare costs from oral disease among older adults.

The lack (or abundance) of information on economic costs due to oral health among older adults can impede (or strengthen) the political will to select and implement programmes to improve oral health. As a result, our objective is to examine the extent to which current research has examined the economic costs from poor oral health among older adults by addressing the following questions:

1. Are there studies available examining the current economic burden of poor oral health among older adults (above age 65) in the United States?
2. What are the components of cost that are included?

## 2 METHODS

We conducted a scoping review by searching PubMed for studies pertaining to the cost of poor oral health among older adults. Scoping reviews are distinct from systematic reviews in that they address broad topics and questions with different research designs, in order to “summarise and disseminate research findings, to identify research gaps, and to make recommendations for future research.”^25^ We adhered to the five‐stage process recommended for scoping reviews.^26^ We ran the search with an end date of December 1, 2019, for English language articles only with no limits on publication type. A detailed PubMed search strategy is provided in Appendix 1. Conceptually, the search query consisted of terms relating to (a) dental disease, (b) cost analyses, and (c) aged or elder adults. We did not incorporate a search of the grey literature using other search engines since we considered it unlikely that grey literature would meet the inclusion criteria.

The inclusion criteria were as follows: studies examining populations representative of the United States; studies focusing on adults ages 65 or older; studies specifically examining oral health conditions among older adults as defined by the CDC (untreated tooth decay, gum disease, tooth loss, oral cancer and chronic disease); studies including cost or economic cost as a reported outcome of interest; quantitative studies; and studies in English or with an English abstract. Specifically, we chose to examine only studies examining populations representative of the United States because the United States has a highly fragmented and complex healthcare system, which impacts how care is accessed and delivered and how much care costs.^27^ In particular, health care spending per capita in the United States is higher than any other nation.^28^ Hence, healthcare cost estimates for diseases coming from other nations would have limited applicability to the US setting.

The exclusion criteria were as follows: studies examining populations outside of the United States; studies focusing on populations outside the included age range; studies not examining oral health conditions specifically; studies not including cost or economic cost as a reported outcome of interest; qualitative studies; and studies not in English or had no English abstract available.

Two independent reviewers (RN and AV) reviewed the titles and the abstracts to assess which publications met the inclusion and exclusion criteria. Any disagreement between the two reviewers was resolved by SH. If an article was determined from the title and abstract to meet the inclusion and exclusion criteria, the full article was then obtained by SH to further examine adherence to the inclusion and exclusion criteria.

We then assessed the full articles for different cost components, as delineated by earlier systematic reviews of cost‐of‐illness studies, which identify the perspective from which costs were calculated,^29^, ^30^, ^31^ what condition was examined and how it was defined in the data,^29^, ^30^, ^31^ and what types of cost categories are included ^30^, ^31^ (ie inpatient care [IP], outpatient medical care [OP], outpatient dental care [Dental], inpatient drugs [IP drugs], outpatient drugs [OP drugs], non‐medical [nursing home, home health visits, physiotherapy, etc], informal care and lost productivity).^29^ For completeness, we also recorded the data collection methods ^29^, ^30^ and data sources.^29^, ^30^, ^31^

## 3 RESULTS

**FIGURE 1:**
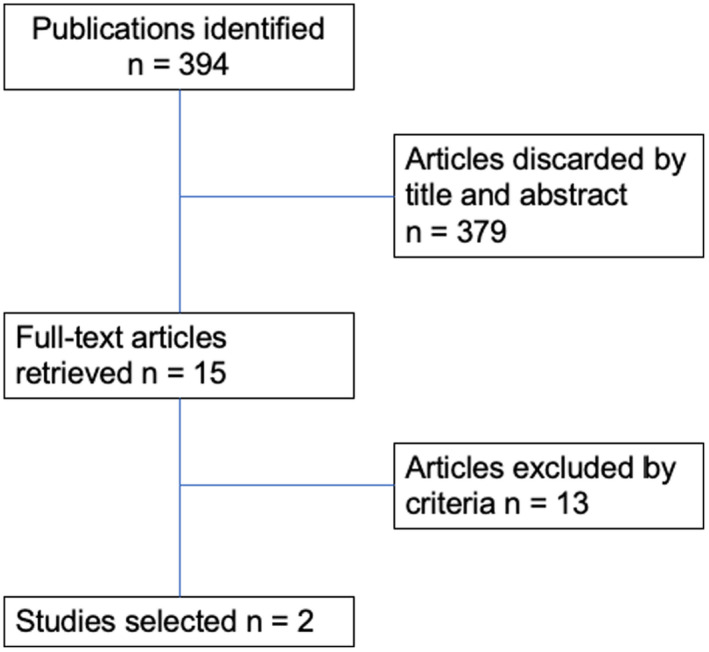
Literature search

The literature review identified 384 abstracts, of which 15 (3.9%) were found to be relevant (Figure 1). When full‐text articles were retrieved for the 15 abstracts found to be relevant, 13 of them were not found to adhere to the inclusion and exclusion criteria. Of the 13 that were deemed not relevant from the full‐text review, 7 articles were found to be based on data for population outside of the United States, 2 articles did not examine the right age group, 2 articles did not focus on specific oral health conditions, and 2 articles were qualitative studies. Ultimately, only 2 articles were deemed relevant (Figure 1).

Both included studies examine components of medical and drug costs for treatment of oral cancer. However, as neither of the studies were cost‐of‐illness studies, the perspective of the cost calculations was not reported. Given that one calculated out‐of‐pocket costs of oral cancer drug therapy using Medicare Part D data,^32^ and the other calculated Medicare inpatient and outpatient costs of oral cancer,^33^ neither one of the studies was comprehensive in including all the costs that would be relevant to Medicare, nor to Medicare beneficiaries for oral cancer. Moreover, there was no reporting of non‐medical care costs, informal care costs, dental costs and lost productivity costs for oral cancer included in either study. The included costs for the two relevant studies in this review are summarised in Table 1.

**TABLE 1:** Included cost categories among studies meeting inclusion and exclusion criteria

	Included Papers	
	Hollenbeak et al 2015	Kaisaeng et al 2014
Condition examined	Oral cavity and pharynx cancers	Oral cancer
Definition of condition	Initial primary tumour of the oral cavity or pharynx diagnosed from Jan 1, 1995 to Dec 31, 2005	Any prescription fill of one of five oral cancer drugs
**Cost Components Included**		
Inpatient medical care costs	Excludes costs related to complications	
Outpatient medical care costs	Excludes costs related to complications	
Inpatient prescription drug costs	Excludes costs related to complications	
Outpatient prescription drug costs		Costs for the 5 top selling oral cancer drugs by sales covered by Part D in 2008 (anastrozole, imatinib, ertinib, letrozole, thalidomide)
Non‐medical care		
Informal care costs		
Dental costs		
Lost productivity costs		
**Study Characteristics**		
Perspective	Not stated	Not stated
Data collection method	Retrospective analysis of 1995‐2005 SEER‐Medicare database	Retrospective analysis of a 5% random sample of 2008 Medicare Part D data
Population	66 years and older receiving cancer diagnosis while alive, FFS patients only	65 years or older at the beginning of 2008, continuously enrolled in Medicare Part D programme from Jan 1, 2008, to Dec 31, 2008; filled prescription for at least one of five oral cancer medications; alive as of Dec 31, 2008; and did not receive Part D prescription subsidies
Sample size	10 711	3781

Other than oral cancer, no studies were found examining the economic costs of poor oral health among older adults (untreated tooth decay, gum disease, tooth loss and chronic disease/s).

## 4 DISCUSSION

Addressing even only the question of how much dental and dental‐related medical utilisation is spent on specific oral disease among older adults is difficult to do in the US setting. This is because comprehensive data capturing oral health, dental utilisation and dental‐related medical utilisation are often difficult to obtain and fragmented, even for a single individual. This is because (a) dental claims databases do not often contain diagnosis codes for dental disease; (b) data on out‐of‐pocket expenses for patients seen without dental insurance are difficult to procure; and (c) dental claims databases are not easily linked to medical claims databases.

Dental diagnosis codes in the United States are seldom used or required when billing for dental procedures, making it difficult to systematically track oral health conditions among older adults. The current diagnosis codes in use are ICD‐10‐CM, which has been in effect since October 1, 2015.^34^ However, the American Dental Association website states that “most dental plans have not announced any intentions to require the use of diagnostic coding for routine dental claims.”^34^ Moreover, it is unclear to which correct and appropriate coding of diagnoses is verifiable among insurers. As a result, tracking specific oral health conditions among older adults is difficult when using dental claims data.

Studies using claims data without dental diagnosis codes or oral health outcomes data are then confined to assessing the cost of procedures associated with a specific disease, but not the cost of disease itself.^20^, ^21^, ^35^ There are a number of scenarios under which cost of disease will differ from the cost of procedures associated with a specific disease under several conditions. One scenario is if procedures are provided before a diagnosis is warranted, either as a prevention measure or overtreatment. Multiple studies have documented that the current fee‐for‐service payment system provides financial incentive for such behaviour.^36^, ^37^, ^38^ In this case, patients may not have a diagnosable disease, but receive treatment that is associated with such disease. Yet, such treatment costs should not be included in the burden of oral disease since disease was not present at the time of treatment. Including these costs will lead to a systematic overestimation of the burden of oral disease. Hence, it is unclear how to reinterpret analyses examining dental interventions' impact on healthcare costs as analyses estimating oral health conditions’ impact on healthcare costs. As a result, studies that examined the impact of specific dental interventions on healthcare costs without defining the oral condition examined were excluded from this scoping review. Future studies should seek to link oral health outcomes data or dental diagnosis codes with dental claims data to avoid this issue.

Assuming however that oral health outcomes data are available or linkable to dental claims data, assessing dental expenditures spent directly on specific oral health conditions among older adults is still difficult. This is because nearly two‐thirds of Medicare beneficiaries have no dental coverage, and more than 35% of adults ages 65 and older with a dental visit paid solely out of pocket without dental insurance.^39^ Data surveying individuals about their dental care utilisation and expenditure that is then linked to dental examinations by calibrated examiners may come closer; however, these data are (a) vulnerable to recall bias and (b) do not capture the share of dental expenditures paid for by dental insurers and the government, unless it is linked to claims data. Dental service organisations, dental schools or other large organisations with multiple dental practices that function using the same billing software (to decrease researchers' burden from data extraction and linkage) likely both (a) have data on dental expenditures by even uninsured patients and (b) have enough data to make data linkage efforts worth it. Future work using these datasets linked with oral health outcomes data would be valuable in this area.

Assessing the impact of oral conditions on medical expenditures carries difficulties as well, even if oral health data are linked to or extrapolated from dental claims data. Dental claims databases are not easily linked to medical claims databases because dental benefits are usually offered as stand‐alone plans separate from medical plans, and usually by different insurers. Linking dental claims and medical claims databases is an onerous task for researchers, though some states have begun collecting comprehensive claims data from both dental and medical insurers (All‐Payer Claims Databases). Yet, there is significant variability in whether the dental and medical databases are linkable at the individual level, since individual identifiers are usually removed from the data to adhere to HIPAA and decrease privacy concerns. However, this may be a potential path for future work as the All‐Payer Claims Databases continue to develop across the states.

However, even with oral health data from dental claims linked to medical claims, it is still likely that potentially avoidable medical costs due to dental disease will be underestimated by researchers. In particular, medical costs for an oral disease may arise when dental care is delayed or not provided, which may mean that oral disease is underdiagnosed. In segments of the population with systematic under‐provision or underuse of dental care, it then becomes difficult to allocate medical costs that should be associated with specific oral diseases. This also means that individuals with dental care, dental diagnoses and dental insurance whose dental claims are linkable to medical claims are systematically different than those with oral disease that are not diagnosed due to lack of dental care. In this case, longitudinal studies that provide oral health examinations for older adults with low dental care access and utilisation to provide dental diagnoses, and that link to their dental and medical claims, may be the only possibility for fully capturing medical costs from otherwise undiagnosed oral disease. Yet such studies also may themselves alter patterns of dental and medical utilisation. Hence, future work that carefully designs studies addressing this gap in the literature is warranted.

A limitation of our approach is that we do not search the grey literature, because we were interested in the peer‐reviewed scientific literature for this initial scoping review. Yet, we believe that this scoping review has established that future systematic reviews in this area would be valuable, which is an important function of scoping reviews.^25^ Future systematic reviews should certainly search the grey literature, especially to ensure coverage of the social science literature.

## 5 CONCLUSION

This review found no studies examining the economic costs of poor oral health among older adults (untreated tooth decay, gum disease, tooth loss and chronic disease/s) in the peer‐reviewed scientific literature outside of oral cancer. Among the two studies found to examine economic costs from oral cancer, neither study sufficiently captured the full range of costs necessary to assess the full economic burden of oral cancer from the US patient, insurer, government and societal perspectives. Future studies are required in the United States to estimate the full economic burden of poor oral health among older adults to inform decision‐making and policymaking regarding dental insurance and dental care for this population.

## CONFLICT OF INTEREST

The authors have no conflicts of interest to declare.

## AUTHORS’ CONTRIBUTIONS

Dr Huang conceived the research idea, conducted data collection, and drafted and critically revised the manuscript. Mr McGowan contributed to the design of the study, conducted data collection and critically revised the manuscript. Dr Veitz‐Keenan contributed to the design of the study, contributed to data analysis and critically revised the manuscript. Dr Niederman contributed to the design of the study, contributed to data analysis and critically revised the manuscript.

## Data Availability

The data supporting this study are available from the corresponding author, SH, upon reasonable request.

## APPENDIX 1

PubMed search strategy:

(("Periodontal Diseases"[MeSH] OR "Periodontitis"[MeSH] OR "Dental Caries"[MeSH] OR "Tooth Loss"[Mesh] OR "Mouth, Edentulous"[Mesh] OR "Tooth Diseases"[Mesh] OR "Mouth Diseases"[Mesh] OR "Dental Caries"[Mesh] OR "poor oral health"[All Fields] OR "bad oral health"[All Fields] OR "poor dental health"[All Fields] OR "poor oral hygiene"[All Fields] OR "poor dental hygiene"[All Fields] OR "Tooth loss"[All Fields] OR "Tooth disease"[All Fields] OR "Tooth diseases"[All Fields] OR "Mouth disease"[All Fields] OR "Mouth diseases"[All Fields] OR "Dental caries"[All Fields] OR "Edentulism"[All Fields]) **AND** ("Costs and Cost Analysis"[Mesh] OR "Costs and Cost Analysis"[All Fields] OR "Cost‐effectiveness studies"[All Fields] OR "Cost‐effectiveness study"[All Fields] OR "Health Care Costs"[Mesh] OR "Economics, Medical"[Mesh] OR "Economic burden"[All Fields] OR "economic analysis"[All Fields] OR "cost analysis"[All Fields] OR "cost of illness"[All Fields]) **AND** ("aged"[MeSH Terms] OR "aged, 80 and over"[MeSH Terms] OR "Frail Elderly"[Mesh] OR "elderly"[All Fields] OR "older adults"[All Fields]))

### OR

(("Costs and Cost Analysis"[Mesh] OR "Costs and Cost Analysis"[All Fields] OR "Cost‐effectiveness studies"[All Fields]

OR "Cost‐effectiveness study"[All Fields] OR "Health Care Costs"[Mesh] OR "Economics, Medical"[Mesh] OR "Economic burden"[All Fields] OR "economic analysis"[All Fields] OR "cost analysis"[All Fields] OR "cost of illness"[All Fields]) **AND** ("Dental Care for Aged"[Mesh]))

English only = 384 ABSTRACTS

## References

[1] Ortman JM, Velkoff VA, Hogan H. An aging nation: the older population in the United States. United States Census Bureau, Economics and Statistics Administration, US. 2014.

[2] Centers for Disease Control and Prevention . Oral Health: Edentulism and Tooth Retention. https://www.cdc.gov/oralhealth/publications/OHSR‐2019‐edentulism‐tooth‐retention.html. Updated September 10, 2019. Accessed May 27, 2020

[3] Ettinger RL. Oral health and the aging population. J Am Dent Assoc. 2007;138:S5‐S6. 10.14219/jada.archive.2007.0357 17761839

[4] Meyerhoefer CD, Zuvekas SH, Farkhad BF, Moeller JF, Manski RJ. The demand for preventive and restorative dental services among older adults. Health Econ. 2019;28(9):1151‐1158. 31264323 10.1002/hec.3921 PMC6706303

[5] Freed M, Neuman T, Jacobson G. Drilling Down on Dental Coverage and Costs for Medicare Beneficiaries. 2019. Kaiser Family Foundation. https://www.kff.org/medicare/issue‐brief/drilling‐down‐on‐dental‐coverage‐and‐costs‐for‐medicare‐beneficiaries/. Updated March 13, 2019. Accessed February 4, 2020.

[6] Hinton E, Paradise J. Access to dental care in Medicaid: spotlight on nonelderly adults. The Henry J Kaiser Family Foundation; 2016.

[7] Griffin SO, Jones JA, Brunson D, Griffin PM, Bailey WD. Burden of oral disease among older adults and implications for public health priorities. Am J Public Health. 2012;102(3):411‐418. 22390504 10.2105/AJPH.2011.300362 PMC3487659

[8] Mirel LB, Carper K. Trends in Health Care Expenditures for the Elderly, Age 65 and Older: 2001, 2006, and 2011. In: Statistical Brief (Medical Expenditure Panel Survey (US))[Internet]. Agency for Healthcare Research and Quality (US). 2014. 29360330

[9] Rosenoer L, Sheiham A. Dental impacts on daily life and stisfaction with teeth in relation to dental status in adults. J Oral Rehabil. 1995;22(7):469‐480. 7562211 10.1111/j.1365-2842.1995.tb01191.x

[10] Sarita PT, Witter DJ, Kreulen CM, Van't Hof MA, Creugers NH. Chewing ability of subjects with shortened dental arches. Commun Dent Oral Epidemiol. 2003;31(5):328‐334. 10.1034/j.1600-0528.2003.t01-1-00011.x 14667003

[11] Idowu A, Handelman S, Graser G. Effect of denture stability, retention, and tooth form on masticatory function in the elderly. Gerodontics. 1987;3(4):161‐164. 3326781

[12] Walls AW, Steele JG, Sheiham A, Marcenes W, Moynihan PJ. Oral health and nutrition in older people. J Public Health Dent. 2000;60(4):304‐307. 11243051 10.1111/j.1752-7325.2000.tb03339.x

[13] Ervin RB, Dye BA. The effect of functional dentition on Healthy Eating Index scores and nutrient intakes in a nationally representative sample of older adults. J Public Health Dent. 2009;69(4):207‐216. 19453869 10.1111/j.1752-7325.2009.00124.x

[14] Ritchie CS, Joshipura K, Silliman RA, Miller B, Douglas CW. Oral health problems and significant weight loss among community‐dwelling older adults. J Gerontol A Biol Sci Med Sci. 2000;55(7):M366‐M371. 10898252 10.1093/gerona/55.7.m366

[15] Sheiham A, Steele J, Marcenes W, Finch S, Walls A. The relationship between oral health status and body mass index among older people: a national survey of older people in Great Britain. Br Dent J. 2002;192(12):703‐706. 12125796 10.1038/sj.bdj.4801461

[16] Sussex PV, Thomson WM, Fitzgerald RP. Understanding the ‘epidemic’ of complete tooth loss among older New Zealanders. Gerodontology. 2010;27(2):85‐95. 19555356 10.1111/j.1741-2358.2009.00306.x

[17] Oakes D, Monopoli M. Medicare dental benefit will improve health and reduce health care costs. In. (Vol 2020) Health Affairs Blog: Health Affairs. 2019.

[18] Albert DA, Begg MD, Andrews HF, et al. An examination of periodontal treatment, dental care, and pregnancy outcomes in an insured population in the United States. Am J Public Health. 2011;101(1):151‐156. 21088265 10.2105/AJPH.2009.185884 PMC3000729

[19] Elani HW, Simon L, Ticku S, Bain PA, Barrow J, Riedy CA. Does providing dental services reduce overall health care costs?: a systematic review of the literature. J Am Dent Assoc. 2018;149(8):696‐703.e692. 29866364 10.1016/j.adaj.2018.03.023

[20] Jeffcoat MK, Jeffcoat RL, Gladowski PA, Bramson JB, Blum JJ. Impact of periodontal therapy on general health: evidence from insurance data for five systemic conditions. Am J Prev Med. 2014;47(2):166‐174. 24953519 10.1016/j.amepre.2014.04.001

[21] Nasseh K, Vujicic M, Glick M. The relationship between periodontal interventions and healthcare costs and utilization. Evidence from an integrated dental, medical, and pharmacy commercial claims database. Health Econ. 2017;26(4):519‐527. 26799518 10.1002/hec.3316 PMC5347922

[22] Clabaugh G, Ward MM. Cost‐of‐illness studies in the United States: a systematic review of methodologies used for direct cost. Value in Health. 2008;11(1):13‐21. 18237356 10.1111/j.1524-4733.2007.00210.x

[23] Tarricone R. Cost‐of‐illness analysis: what room in health economics? Health Policy. 2006;77(1):51‐63. 16139925 10.1016/j.healthpol.2005.07.016

[24] Segel JE. Cost‐of‐illness studies—a primer. RTI‐UNC Center of Excellence in Health Promotion Economics. 2006;1:39.

[25] Peters MD, Godfrey CM, Khalil H, McInerney P, Parker D, Soares CB. Guidance for conducting systematic scoping reviews. JBI Evidence Implementation. 2015;13(3):141‐146. 10.1097/XEB.0000000000000050 26134548

[26] Peters M, Godfrey C, Khalil H. The Joanna Briggs Institute Reviewers’ manual: methodology for JBI scoping review. Australia: Adelaide; 2015.

[27] Anderson GF, Reinhardt UE, Hussey PS, Petrosyan V. It’s the prices, stupid: why the United States is so different from other countries. Health Aff. 2003;22(3):89‐105. 10.1377/hlthaff.22.3.89 12757275

[28] Papanicolas I, Woskie LR, Jha AK. Health care spending in the United States and other high‐income countries. JAMA. 2018;319(10):1024‐1039. 29536101 10.1001/jama.2018.1150

[29] Quentin W, Riedel‐Heller S, Luppa M, Rudolph A, König HH. Cost‐of‐illness studies of dementia: a systematic review focusing on stage dependency of costs. Acta Psychiatr Scand. 2010;121(4):243‐259. 19694634 10.1111/j.1600-0447.2009.01461.x

[30] Luppa M, Heinrich S, Angermeyer MC, König H‐H, Riedel‐Heller SG. Cost‐of‐illness studies of depression: a systematic review. J Affect Disord. 2007;98(1–2):29‐43. 16952399 10.1016/j.jad.2006.07.017

[31] Dagenais S, Caro J, Haldeman S. A systematic review of low back pain cost of illness studies in the United States and internationally. Spine J. 2008;8(1):8‐20. 18164449 10.1016/j.spinee.2007.10.005

[32] Kaisaeng N, Harpe SE, Carroll NV. Out‐of‐pocket costs and oral cancer medication discontinuation in the elderly. J Manag Care Spec Pharm. 2014;20(7):669‐675. 24967520 10.18553/jmcp.2014.20.7.669 PMC10437384

[33] Hollenbeak CS, Kulaylat AN, Mackley H, Koch W, Schaefer EW, Goldenberg D. Determinants of medicare costs for elderly patients with oral cavity and pharyngeal cancers. JAMA Otolaryngology‐Head Neck Surg. 2015;141(7):628‐635. 10.1001/jamaoto.2015.0940 26042925

[34] American Dental Association . Answers to Frequently Asked Questions About ICD‐10‐CM. 2015. Updated October 1, 2015. Accessed February 10, 2020.

[35] Medical Dental Integration Study. UnitedHealthcare. In: 2013.

[36] Chalkley M, Listl S. First do no harm–The impact of financial incentives on dental X‐rays. Journal of health economics. 2018;58:1‐9. 29408150 10.1016/j.jhealeco.2017.12.005

[37] Grytten J. Supplier inducement–its relative effect on demand and utilization. Commun Dent Oral Epidemiol. 1992;20(1):6‐9. 10.1111/j.1600-0528.1992.tb00664.x 1547616

[38] Niederman R, Huang SS, Trescher A‐L, Listl S. Getting the incentives right: improving oral health equity with universal school‐based caries prevention. Am J Public Health. 2017;107(S1):S50‐S55. 28661798 10.2105/AJPH.2016.303614 PMC5497868

[39] Nasseh K, Vujicic M. Dental Care Utilization Rate Highest Ever Among Children, Continues To Decline Among Working‐Age Adults. Health Policy Institute Research Brief Illinois: American Dental Association; 2014.

